# Endobronchial Aspergillosis Presenting as an Isolated Airway Lesion in an Immunocompromised Patient

**DOI:** 10.1002/rcr2.70701

**Published:** 2026-09-01

**Authors:** Mohsen Sadeghi, Fatemeh Sadat Hosseini Khajouei, Haniyeh GhanbarAliAkhavan, Amir Morteza Soleimani

**Affiliations:** ^1^ Chronic Respiratory Diseases Research Center (CRDRC), National Research Institute of Tuberculosis and Lung Diseases (NRITLD), Shahid Beheshti University of Medical Sciences Tehran Iran; ^2^ Inflammatory Lung Disease Research Center, Department of Internal Medicine, Razi Hospital, School of Medicine Guilan University of Medical Sciences Rasht Iran; ^3^ Student Research Committee Arak University of Medical Sciences Arak Iran; ^4^ Department of Internal Medicine, School of Medicine Arak University of Medical Sciences Arak Iran; ^5^ Clinical Research Development Unit of Amiralmomenin Hospital Arak University of Medical Sciences Arak Iran

**Keywords:** airway lesion, endobronchial aspergillosis, fungal airway infection, immunocompromised host

## Abstract

Endobronchial aspergillosis (EBA) is an uncommon manifestation of *Aspergillus* airway disease characterized by fungal involvement of the tracheobronchial lumen. It may present with non‐specific respiratory symptoms, including dyspnea, cough and hemoptysis, and can mimic endoluminal airway tumours. Predisposing factors include immunosuppressive conditions such as corticosteroid therapy, malignancy, chemotherapy and neutropenia. Definitive diagnosis relies on bronchoscopic evaluation with histopathological confirmation. Due to its rarity, evidence regarding optimal management remains limited. This case emphasizes that isolated airway *Aspergillus* infection may occur without parenchymal lung involvement and may be associated with a rapidly fatal clinical course despite prompt antifungal therapy.

## Graphical abstract

Endobronchial *Aspergillus* infection should be considered in immunocompromised patients presenting with airway obstruction, even in the absence of parenchymal lung involvement. This case highlights the potential for rapid clinical deterioration and the diagnostic challenges in distinguishing endobronchial aspergillosis from more aggressive forms of *Aspergillus* tracheobronchial disease.

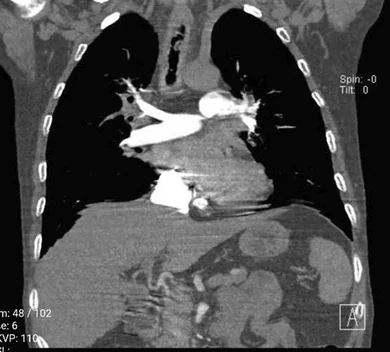

## 1 Introduction

Pulmonary aspergillosis represents the most common fungal infection involving the lungs and predominantly affects immunocompromised individuals, including those receiving corticosteroids, patients with malignancies undergoing chemotherapy, individuals with hematologic disorders, organ transplant recipients, patients with Human Immunodeficiency Virus (HIV) infection, and those with concurrent infections such as influenza or SARS‐CoV‐2 [1]. Herein, we report an unusual case of isolated endotracheal *Aspergillus* infection in a patient with DLBCL and profound chemotherapy‐related neutropenia. Unlike the more common presentations of pulmonary aspergillosis, no radiologic evidence of parenchymal lung involvement was identified. The disease manifested as a solitary obstructing tracheal lesion and was associated with rapidly progressive respiratory failure and death despite prompt antifungal therapy. This case highlights the diagnostic challenges and potentially fulminant course of *Aspergillus* airway disease in severely immunocompromised patients.

## 2 Case Report

A 54‐year‐old man with a history of DLBCL undergoing chemotherapy (last cycle 6 months prior to presentation) was admitted with progressive dyspnea, productive cough and intermittent non‐massive hemoptysis. He denied fever, smoking history or recent immobilisation.

On physical examination, vital signs were stable and oxygen saturation was within normal limits. Mild crackles were auscultated over the lungs.

Laboratory evaluation revealed anaemia and severe leukopenia with marked neutropenia (absolute neutrophil count [ANC]: 150/μL). Serum biochemistry and arterial blood gas analysis were unremarkable.

**FIGURE 1:**
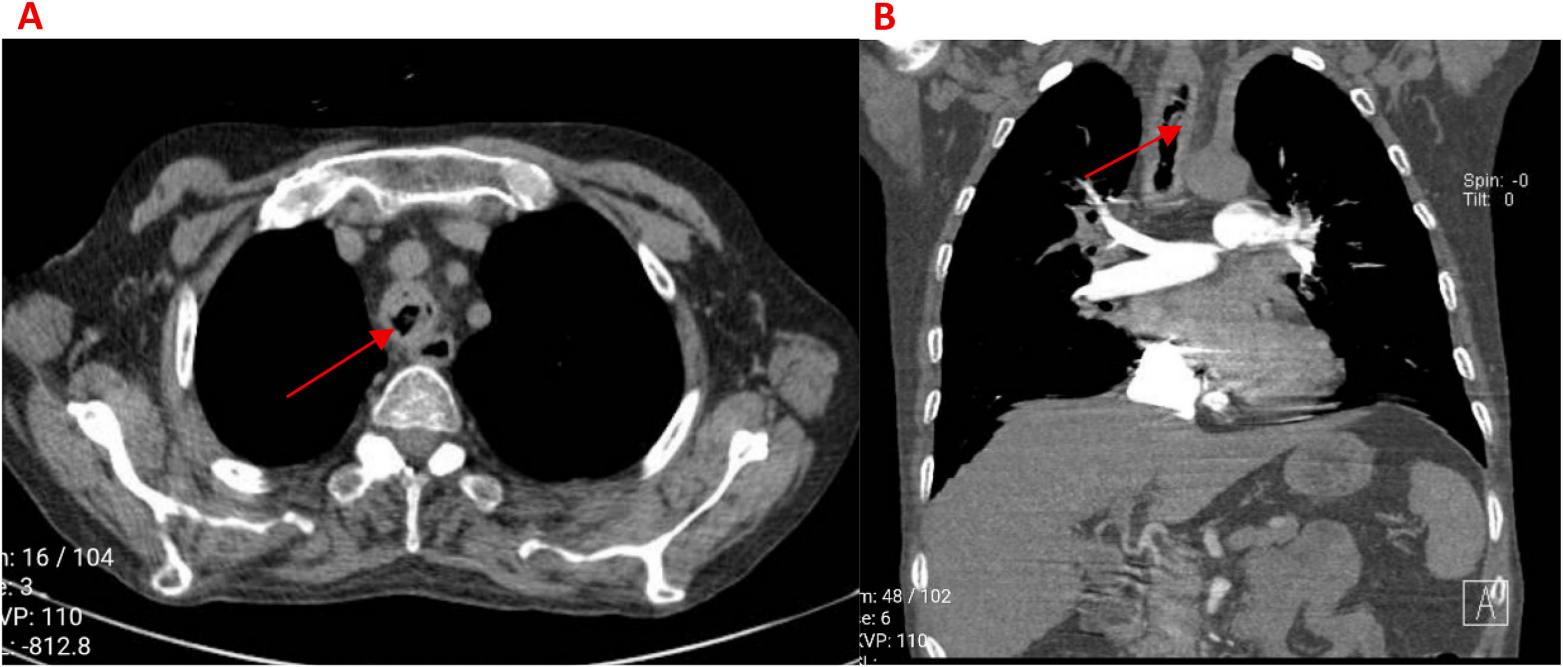
Coronal and axial chest CT images showing an intratracheal mass‐like lesion at the level just inferior to the suprasternal notch, with no evidence of associated pulmonary parenchymal involvement.

Chest computed tomography (CT) scan demonstrated a mass‐like lesion along the posterior wall of the trachea, just inferior to the suprasternal notch, without evidence of parenchymal lung involvement (Figure 1).

**FIGURE 2:**
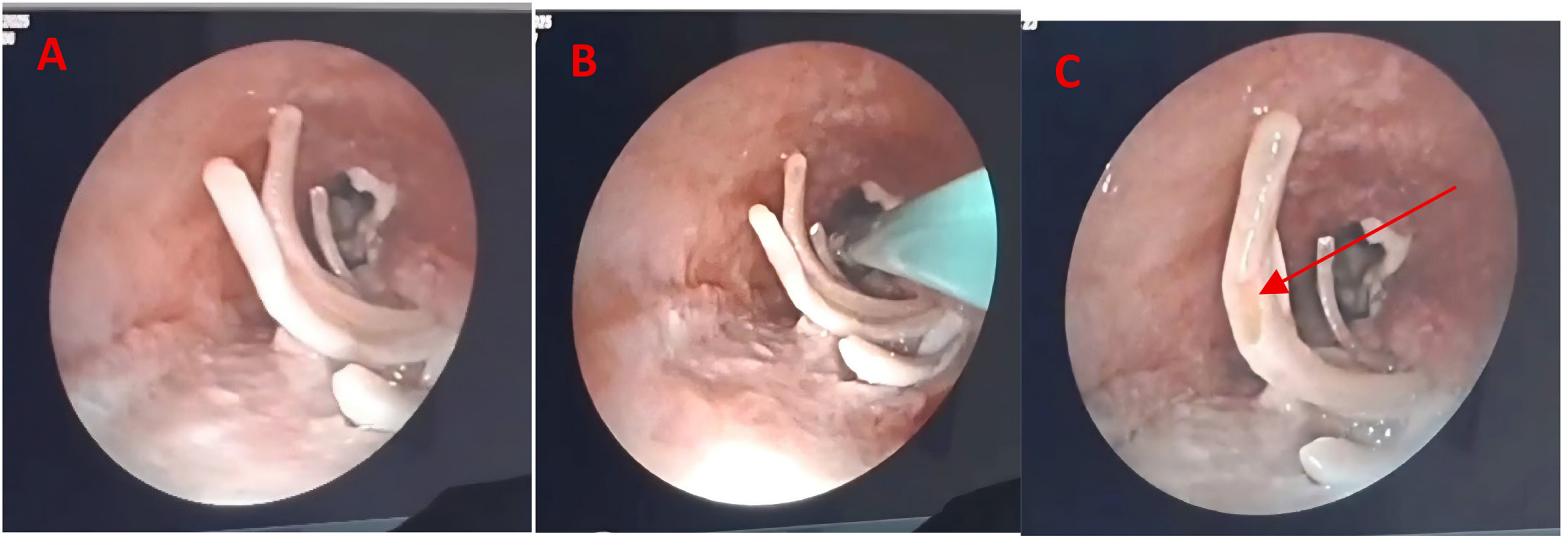
(A) Flexible bronchoscopy demonstrating a whitish, irregular endotracheal lesion with pseudomembranous and filamentous characteristics, without evidence of mucosal invasion or active bleeding. (B) Endobronchial biopsy forceps used for sampling of the tracheal lesion under flexible bronchoscopic guidance. (C) Post‐biopsy bronchoscopic image of the lesion site, showing the biopsy area following tissue sampling, without active bleeding or apparent airway injury.

Flexible bronchoscopy revealed a whitish, irregular endoluminal mass with elongated, intertwined projections and a pseudomembranous appearance located in the mid‐trachea. The lesion caused significant luminal obstruction, precluding advancement of the bronchoscope distally. No overt mucosal invasion, necrosis or active bleeding was observed (Figure 2).

**FIGURE 3:**
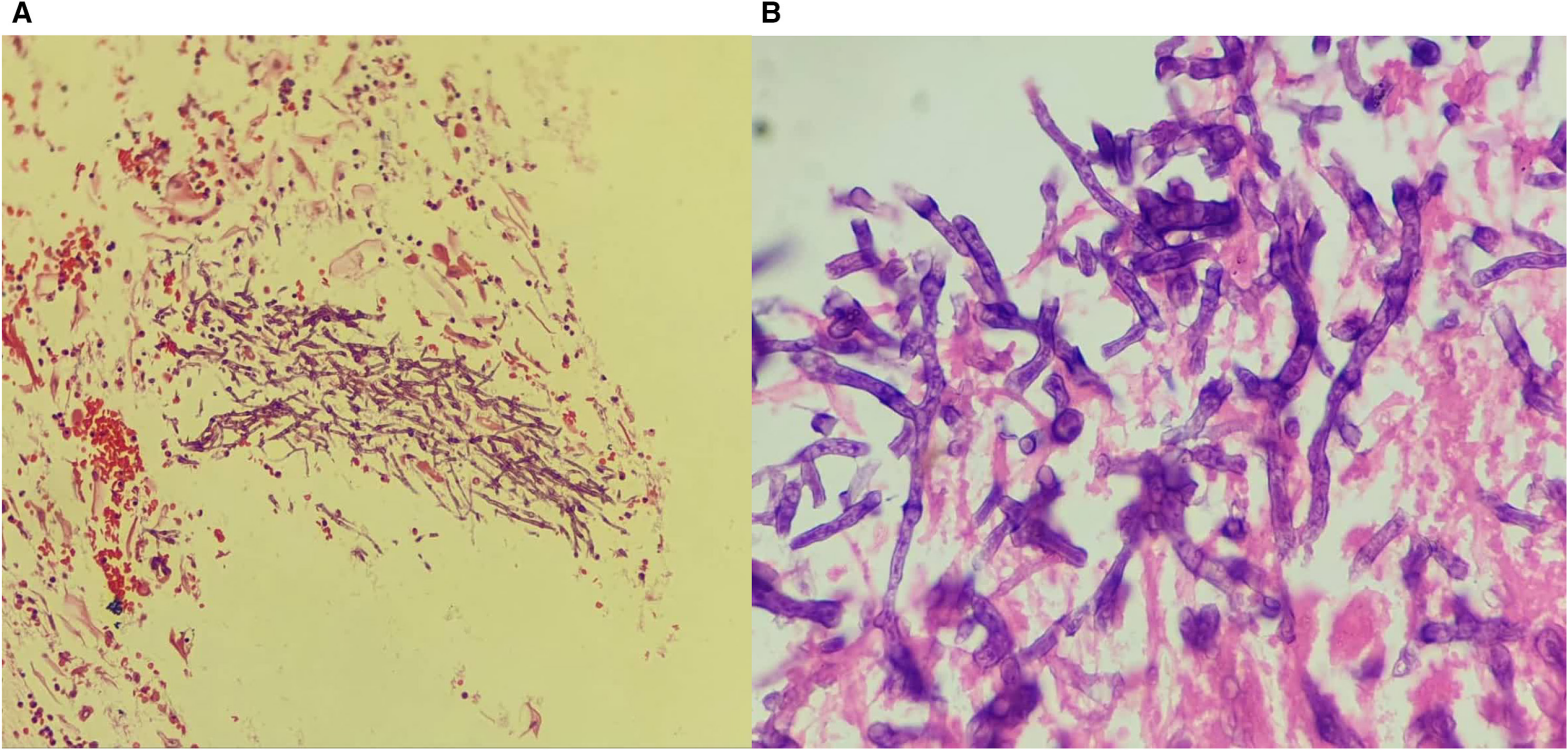
Histopathological examination revealing septate, hyaline hyphae with acute‐angle (≈45°) branching, consistent with *Aspergillus* species morphology.

Endobronchial samples were obtained for microbiological and histopathological analysis. Microscopy demonstrated septate hyphae with acute‐angle branching (Figure 3). Polymerase chain reaction confirmed *Aspergillus fumigatus* infection.

Despite initiation of antifungal therapy with amphotericin B and voriconazole, the patient's clinical condition deteriorated rapidly, and he died within 36 h due to respiratory failure.

The exact mechanism of the patient's rapid clinical deterioration and respiratory failure remains uncertain. Bronchoscopy demonstrated a significant obstructing tracheal lesion that precluded advancement of the bronchoscope beyond the site of obstruction. Consequently, distal airway involvement, mucus impaction or more extensive tracheobronchial disease could not be excluded. Given the profound neutropenia and fulminant clinical course, multiple factors may have contributed to the fatal outcome.

## 3 Discussion

*Aspergillus* is a genus of filamentous fungi that exists in two morphological forms: spores (conidia) and hyphae [2]. Among its species, fumigatus, flavus, niger, terreus, nidulans and calidoustus are responsible for human infections, with *Aspergillus fumigatus* accounting for more than 90% of aspergillosis cases [3, 4, 5]. *Aspergillus* species exhibit no specific geographic predilection and are ubiquitously found in water, food, air, soil, and particularly in decaying vegetation [2]. Infection typically occurs via inhalation of airborne conidia into the host's respiratory tract; however, transmission through inhalation of aerosolized water contaminated with conidia has also been reported [6, 7]. Consequently, the lungs are the most commonly affected organ in aspergillosis, reflecting the primary route of spore entry into the airways [8]. Notably, *A. fumigatus* demonstrates greater adherence to airway (AW) epithelium compared to other species [1, 2].

Pulmonary aspergillosis comprises several clinical entities, including aspergilloma, allergic bronchopulmonary aspergillosis (ABPA), chronic pulmonary aspergillosis and invasive pulmonary aspergillosis (IPA), each reflecting a distinct interaction between the host immune status and *Aspergillus* species [4]. Among these, IPA is the most severe form and occurs predominantly in immunocompromised individuals, particularly those with prolonged neutropenia or hematologic malignancies [4, 9].

Endobronchial aspergillosis (EBA) is a rare manifestation of *Aspergillus* airway disease characterized by endoluminal fungal growth within the tracheobronchial tree [10, 11]. In 2019, Ngu et al. [12] reported that only 28 cases of EBA had been documented worldwide, most occurring in Asian males with a mean age of 58 years [12]. Similarly, Huang et al. described 17 cases with comparable demographic characteristics [13].

EBA is often diagnosed incidentally during bronchoscopy performed for other indications, such as evaluation of hemoptysis. Clinical manifestations may include fever, dyspnea, chest pain, cough and hemoptysis [11]. Risk factors include any form of immunosuppression as well as underlying pulmonary disease [14].

Neutrophils constitute the first line of defence against fungal invasion [15, 16]; therefore, neutropenia is a major predisposing factor for *Aspergillus* infection. An ANC below 500/μL or prolonged neutropenia exceeding 20 days represents one of the strongest risk factors [9], with the risk rising to approximately 70% after 34 days of persistent neutropenia [17].

Ma et al. reported 10 patients with EBA in 2011, all of whom had underlying risk factors such as tuberculosis, lung cancer or foreign body aspiration; concomitant parenchymal involvement was observed in only two cases [11]. Similarly, our patient had severe chemotherapy‐induced neutropenia (ANC: 150/μL) without evident parenchymal lung involvement.

Due to airway obstruction caused by endobronchial fungal proliferation, EBA may progress to respiratory failure [18, 19]. Patients requiring mechanical ventilation have significantly higher mortality compared to those who do not (94% vs. 25%), reflecting advanced disease and poor prognosis [20].

Radiological diagnosis of EBA is challenging, as findings may mimic foreign bodies or endobronchial tumours [13]. Definitive diagnosis is typically established via bronchoscopic biopsy and histopathological evaluation. Bronchoscopic findings range from white–yellow necrotic plaques to nodular or infiltrative lesions involving the airway mucosa [21].

A limitation of the present case is that histopathological assessment was confined to the obstructing endoluminal lesion. Because of the patient's critical condition and the potential risk of procedure‐related complications, no biopsy specimens were obtained from the tracheal wall or cartilage. Therefore, direct evidence of mucosal, submucosal or cartilaginous invasion was unavailable. Although the bronchoscopic appearance, profound neutropenia, and rapidly progressive clinical course raised concern for a more aggressive form of *Aspergillus* tracheobronchial disease, the degree of airway wall invasion could not be definitively determined.

The present case also raises important diagnostic considerations regarding the spectrum of *Aspergillus* tracheobronchial disease. The pseudomembranous bronchoscopic appearance, profound neutropenia, and rapidly fatal clinical course may suggest a more aggressive form of *Aspergillus* tracheobronchitis. However, because histopathological evaluation was limited to the endoluminal lesion and no airway wall biopsy was obtained, definitive classification of the lesion as invasive or necrotizing tracheobronchitis was not possible.

The diagnostic gold standard for EBA is fungal culture and isolation of the organism [10]. However, this approach is limited by challenges such as difficulty in obtaining adequate samples in hemodynamically unstable patients, as well as coagulopathies like thrombocytopenia. Furthermore, the sensitivity of culture varies depending on specimen type, quality and timing of collection [22, 23]. Consequently, indirect diagnostic methods—such as detection of galactomannan in serum or bronchoalveolar lavage (BAL) and polymerase chain reaction (PCR)‐based assays—have been developed [24].

False‐negative culture results may occur due to prior or ongoing antifungal therapy [25]. Galactomannan testing demonstrates reasonable reliability in patients with hematologic malignancies, although its sensitivity ranges from 44% to 90% [26, 27]. False‐positive results have been reported in patients receiving antibiotics such as piperacillin–tazobactam or amoxicillin–clavulanate, as well as in infections caused by dimorphic fungi including Histoplasma capsulatum and Blastomyces dermatitidis [28]. Measurement of β‐D‐glucan is of limited utility due to its lack of specificity for aspergillosis [29].

Currently, no standardized or optimal treatment strategy for EBA has been established, and management is individualized [30]. Most clinicians advocate a combination of systemic antifungal therapy and interventional bronchoscopy [30]. In certain cases, intolerance or resistance to azoles or the presence of concomitant infections, may necessitate surgical resection. Notably, Ma et al. adopted a conservative approach and did not administer antifungal therapy, arguing that systemic antifungal agents may have limited penetration into airway lesions [12].

Argento et al. reported favourable outcomes using a combined approach of systemic antifungal therapy and bronchoscopically guided resection of the endobronchial lesion [31].

To our knowledge, isolated obstructing tracheal *Aspergillus* lesions without radiologic evidence of parenchymal involvement are exceedingly uncommon. The combination of profound neutropenia, isolated airway disease and a rapidly fatal clinical course makes the present case particularly noteworthy.

It should be noted that liposomal amphotericin B is associated with lower nephrotoxicity compared to amphotericin B deoxycholate. Adverse effects such as renal tubular acidosis (RTA), hypomagnesemia and hypokalemia have shifted clinical preference toward azoles and echinocandins in the management of pulmonary aspergillosis.

In conclusion, endobronchial *Aspergillus* infection should be considered in immunocompromised patients presenting with airway obstruction, even in the absence of parenchymal lung involvement. This case highlights the potential for rapid clinical deterioration and the diagnostic challenges in distinguishing EBA from more aggressive forms of *Aspergillus* tracheobronchial disease.

## Author Contributions

**Mohsen Sadeghi:** supervision, writing – original draft, writing – review and editing. **Haniyeh GhanbarAliAkhavan:** data curation, writing – original draft. **Fatemeh Sadat Hosseini Khajouei:** writing – review and editing. **Amir Morteza Soleimani:** conceptualization, investigation, methodology, project administration, writing – review and editing.

## Funding

The authors have nothing to report.

## Ethics Statement

Ethical approval was not required for this case report according to institutional policies of Arak University of Medical Sciences. This study was conducted in accordance with the principles of the Declaration of Helsinki.

## Consent

The authors declare that written informed consent for publication of this manuscript and the accompanying images was obtained from the patient's next of kin (the patient's brother), as the patient had passed away prior to manuscript preparation. The authors attest that the consent procedure and documentation comply with the Journal's requirements as outlined in the author guidelines.

## Conflicts of Interest

The authors declare no conflicts of interest.

## Data Availability

The data that support the findings of this study are available on request from the corresponding author. The data are not publicly available due to privacy or ethical restrictions.
